# A Case of Oral Health Management for a Patient with Extensive Ulceration of the Oral Mucosa Due to Herpes Zoster

**DOI:** 10.3390/healthcare10112249

**Published:** 2022-11-10

**Authors:** Yuhei Matsuda, Ruriko Mizuno, Saki Miyajima, Shinichi Arakawa, Yuji Kabasawa

**Affiliations:** 1 Tokyo Medical and Dental University Hospital, Oral Health Center, Tokyo 113-8501, Japan; 2 Department of Lifetime Oral Health Care Sciences, Graduate School of Medical and Dental Sciences, Tokyo Medical and Dental University, Tokyo 113-8501, Japan; 3 Department of Oral Care for Systemic Health Support, Graduate School of Medical and Dental Sciences, Tokyo Medical and Dental University (TMDU), Tokyo 113-8510, Japan

**Keywords:** herpes zoster, oral health management, extensive ulceration, oral care, oral mucosa

## Abstract

Oral ulcers caused by herpes zoster virus infection are commonly encountered in daily clinical practice. However, in rare cases, sepsis or viremia can occur with serious outcomes; hence, these must be managed effectively. Here, we report a case of a patient with extensive ulceration caused by varicella zoster virus infection. Antiviral treatment was started early, and oral health management was started simultaneously, with oral hygiene instructions for pain control. As a result, the patient was able to resume oral food intake and was discharged from the hospital within a week. This case suggested that oral health management comprising supportive care, with the assistance of dentists and dental hygienists, as well as antiviral therapy, are important in the treatment of oral ulcers associated with herpes zoster virus infection.

## 1. Introduction

Pathologies of the palate present a major discomfort for patients. Pathologies of the soft tissue of the palate include mucosal wounds, schwannoma of the palate, vascular anomalies, lipoma, oral fibroma, and viral infections, such as the herpes zoster or human papillomavirus [1,2,3,4,5]. Herpes zoster occurs when the varicella-zoster virus (VZV) becomes latent in the dorsal root ganglia of the spinal cord and trigeminal ganglia and is reactivated by immunosuppression or other mechanisms. In Japan, with an increase in its aging population, the number of patients with autoimmune diseases, including rheumatoid arthritis, is increasing, and VZV reactivation may occur due to steroid use [6]. The symptoms and findings of this condition are characteristic painful vesicles that appear along the area innervated by the ganglion, along with blisters and ulcers in the oral cavity [7]. The treatment of herpes zoster primarily involves prevention of complications, prevention of infection in immunocompromised patients, and pain control. Administration of antiviral drugs in the early stages of VZV infection is efficacious to some degree, but complete control of post-VZV neuralgia is difficult to achieve [8]. An epidemiological study of VZV infections in Japan revealed a mean incidence rate of 4.15/1000 person years, with women having a significantly higher incidence rate than men [9]. The primary treatment involves the administration of antiviral drugs (acyclovir, valacyclovir, or famciclovir) within 72 h of the onset of skin rash [10]. For pain control, acetaminophen and non-steroidal anti-inflammatory drugs (NSAIDs) are used in mild cases, while opioids and tricyclic antidepressants should be considered in severe cases [11]. In the absence of an assessment tool for the ulcers, the Common Terminology Criteria for Adverse Events (CTCAE) ver. 5.0, a standard for evaluating oral adverse events in cancer treatment, can be utilized [12]. Although there are some guidelines for therapy for herpes zoster infection, there are no case reports detailing extensive ulceration of the oral cavity due to VZV infection. The purpose of this case presentation is to highlight the need for oral management of VZV patients with extensive ulcers.

## 2. Case Presentation

This case report was approved by the Ethics Committee of Tokyo Medical and Dental University’s Faculty of Dentistry (D2018-016).

### 2.1. Case History

The patient was a 75-year-old woman with a history of rheumatoid arthritis who had been treated with methylprednisolone, prednisolone, and iguratimod. In May 2022, the patient experienced headache and fever and had a skin rash on the left side of her face; she visited the local doctor who diagnosed herpes zoster and administered famciclovir. The next day, the patient was urgently admitted to Tokyo Medical and Dental University Hospital’s Department of Dermatology because of ulceration in the oral cavity and difficulty in oral intake; treatment with acyclovir was initiated. On day 2 following admission, the patient was referred to Tokyo Medical and Dental University’s Oral Health Center for oral health management.

### 2.2. Initial Oral Evaluation

**Figure 1:**
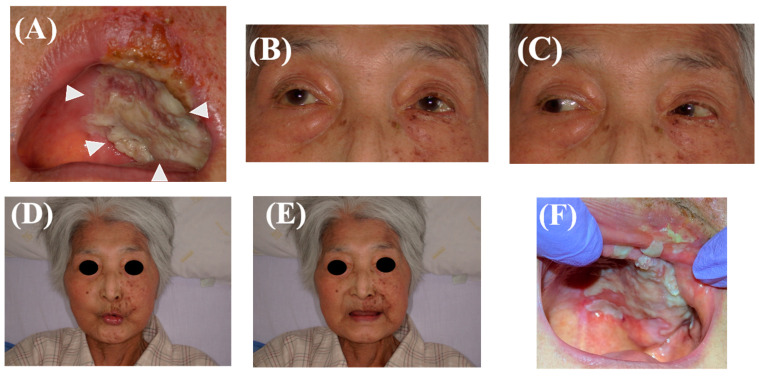
(**A**) Ulcers of the oral cavity and lips, and extensive spread of herpes to the buccal skin. Photograph of facial appearance and oral cavity at the initial visit. (**B**) No ocular symptoms (keratitis, iris ciliary inflammation, scleritis) due to herpes zoster virus infection in the region of the first branch (V1) of the trigeminal nerve. (**C**) No eye movement disorders. (**D**) No abnormal findings in facial nerves that move facial muscles to purse mouth, pull lips, (**E**) raise eyebrows, etc. (**F**) Photograph of facial appearance and oral cavity 6 days later.

Multiple blisters on the palate had self-destructed and fused to form a large ulcer. The patient had Grade 2–3 oral mucositis, as assessed based on the evaluation criteria for oral mucositis caused by anticancer drug therapy [12]. The patient complained of a large ulcer (50 mm × 70 mm), which was located on the left hard palate with a dark red center and surrounding white tissue; there was a vesicular bullae on the buccal skin along the second branch of the left trigeminal nerve (maxillary nerve). The ulceration on the palate was extensive and might have invaded the connective tissue (Figure 1A); however, there was concern about necrosis of the palatal tissue due to the lack of blood flow. Since no paralysis of the facial nerve or the oculomotor, glenoid, or abducens nerves was detected, we initiated an oral health management intervention for herpes zoster virus infection confined to the oral cavity and cheek area (Figure 1B–E). The dentist asked the dental hygienist to provide oral health management to improve oral hygiene and help restore oral intake.

### 2.3. Oral Hygiene Management

On day 1 of intervention at the Tokyo Medical and Dental University Oral Health Center, the patient rinsed her mouth with sodium azulenesulfonate containing lidocaine as a measure against contact pain associated with tooth brushing; then, the oral cavity was scrubbed and cleaned with a sponge brush. The dental hygienist also instructed a nurse about this method to ensure continuity of care. Since the patient was delirious, oral cleaning by a dental hygienist was performed every 2 days without patient intervention. On day 4 of the intervention, pain subsided, and the consciousness level improved. Therefore, the patient initiated self-care, and dental hygiene instructions were provided to the patient. On day 6 of intervention, granulation tissue growth around the ulcers was observed, contact pain had disappeared, and the skin rash on the cheek changed into crust tissue (Figure 1F). The patient was able to eat half a bowl of gruel and was discharged home on day 7 of the intervention. Since the patient had an edentulous maxilla and mandible and could not wear dentures for some time, whole gruel, which does not require mastication, was selected as the food of choice. The patient was instructed about the timing of denture resumption after consultation with the dentist; the dentures could be used only after confirming the lack of pain while wearing them and the ulcer no longer being detectable by the patient.

### 2.4. Follow-Up after Discharge

**Figure 2:**
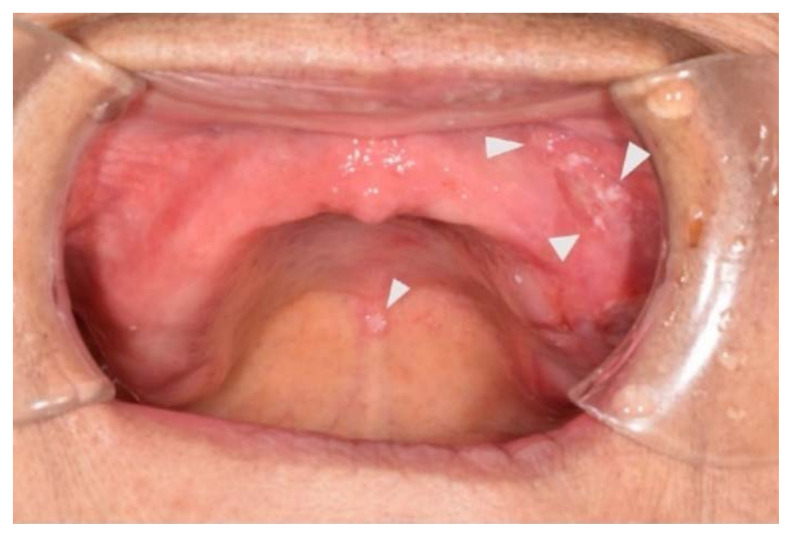
Oral findings 19 days after discharge. Ulcers in the oral cavity epithelialized and disappeared. White tongue coating spreading into the oral cavity and a finding of oral candidiasis.

**Figure 3:**
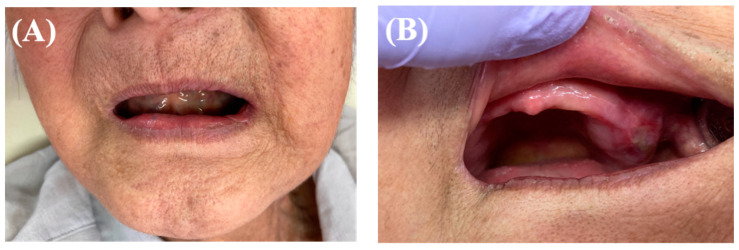
Oral and facial findings 30 days after discharge. (**A**) Blisters on the cheeks disappeared. (**B**) Ulcers in the oral cavity epithelialized and completely disappeared (denture ulcers remained).

The patient lived with a family member who assisted the patient by preparing meals for her. All foods were supposed to be chopped finely to assist the patient in ingestion without wearing dentures until the ulcer disappeared. Upon follow-up at the Tokyo Medical and Dental University Oral Health Care Center on day 19 after intervention, the ulcer had disappeared; however, since there was still a finding of oral candidiasis, the follow-up was extended, and the patient was provided oral cleaning instructions and prescribed antifungal medication (Figure 2). By day 30 post-intervention, all oral lesions had completely resolved, and the patient had recovered to the point of oral intake of a regular diet (Figure 3A,B).

### 2.5. Treatment Result

With VZV reactivation, the patient had experienced difficulty in oral intake due to extensive ulcers in the oral cavity. Following antiviral medication and oral health management, the patient was able to resume oral intake at an early stage and was discharged after 8 days of hospitalization. Within approximately a month, almost all complications resolved. Oral health management was considered to be a healing facilitator rather than a fundamental treatment.

## 3. Discussion

There were concerns about complications from VZV infection (meningitis, myelitis, Ramsay Hunt syndrome); however, these did not occur in this case. Secondary complications include deterioration of extensive oral ulcers and sepsis due to the oral commensal bacteria from the ulcers invading the blood vessels. There has been a case report in Japan of a patient receiving immunosuppressive drugs (such as methotrexate as a treatment for rheumatoid arthritis) who suffered from shingles and died of viremia caused by an ulcer that developed on his lower limb [13]. A descriptive epidemiologic study of 62 children with herpes zoster noted that the prevalence of oral symptoms, such as ulcers, may be associated with the severity of herpes zoster [14]. Therefore, our concerns about the patient developing sepsis and viremia might be accorded with reason, and prevention of secondary infections as well as radical treatment with antiviral drugs seemed important.

To the best of our knowledge, there are no reports on the methods of oral care for extensive ulcers caused by VZV. Therefore, we conducted an intervention according to the method of oral care for oral mucositis associated with cancer treatment [15]. The grade of oral ulcer was estimated to be Grade 2–3 according to CTCAE, version 5.0, because multiple ulcers without spontaneous bleeding were fused, and there was severe pain [16]. The objectives of oral health management were, first, to restore oral function limited by pain, and second, to prevent secondary infections (sepsis and viremia) from oral ulcers due to poor oral hygiene. The use of NSAIDs as a base for VZV-related neuralgia has been reported to be ineffective; thus, acetaminophen and opioids were used as the base pain controllers in this case [17]. For the contact pain of oral ulcers caused by shingles, a mouthwash containing lidocaine, which is known to be effective for short-term pain relief of oral mucositis caused by radiotherapy for head and neck cancer, was also used [18]. The patient was able to resume oral intake due to regular and early initiation of use of a mouthwash containing lidocaine before painful activities (oral cleaning and eating), as per our instructions. Although Episil^®^ oral liquid was not prescribed in this case because the Japanese insurance system does not allow its use, it should be considered as a strategy for pain control [19]. For oral hygiene management, rinsing with mouthwash containing azulene sulfonic acid and physical cleaning of the oral mucosa by rubbing with a sponge brush were performed for pain control [20]. Since oral dryness has been noted to increase the pain of oral mucositis, we also instructed the patient to use moisturizers [21]. The above interventions were considered to be successful and contributed to the prevention of secondary infection in our patient. In Japan, dentists and dental hygienists have been increasingly involved in cancer treatment in recent years [22]. Through this case study, it is suggested that the establishment of a system in which dental professionals collaborate not only in cancer treatment but also in the treatment of other systemic diseases would contribute to improving the quality of treatment. Since this is only a report of one case, further research is needed to determine the effectiveness of oral health management.

## 4. Conclusions

We were able to successfully treat a patient with extensive ulceration caused by herpes zoster virus infection by implementing oral hygiene management focused on pain control and dental hygiene instruction considering the patient’s general condition. The importance of oral hygiene as well as antiviral medications was indicated. The case also suggested the importance of multidisciplinary cooperation in oral hygiene management.

## Data Availability

Not applicable.
